# An overlooked risk for skin health: Less availability and higher cost of sunscreen for people with melanated skin

**DOI:** 10.1016/j.dialog.2024.100194

**Published:** 2024-09-23

**Authors:** Emerson D. Basch, Grace C. Hillyer

**Affiliations:** a Avenues: The World School, New York, NY, USA; b Department of Epidemiology, Mailman School of Public Health, Columbia University, New York, NY, USA

**Keywords:** Skin cancer, Sunscreen, Melanoma, Cancer prevention, Melanated skin, New York City

## Abstract

**Background:**

Sunscreen with a skin protection factor (SPF) of 30 or greater is recommended to reduce the risk of skin cancer and improve skin health for all people regardless of skin tone. Traditional sunscreen that creates a white cast on the skin is incompatible with melanated skin.

**Methods:**

The number of products on the shelf, SPF level, application, coloration, and cost were recorded at three beauty supply chain stores and three pharmacy, health, and wellness stores in or near Harlem in New York City in the Spring of 2023. The number of skin tones for tinted sunscreen was visually matched to the Fitzpatrick Skin Phototypes scale.

**Results:**

A total of 385 sunscreen products were identified; 78.7 % were traditional white sunscreen, followed by sheer (15.3 %) and tinted (6.0 %) products. Beauty supply stores offered more tinted sunscreen options than did pharmacy, health, and wellness stores (*n* = 17,15.3 % vs. *n* = 6, 2.2 %, *p* < 0.001). Of the tinted sunscreen products, 19 brands offered only a single tone. Tinted sunscreen was significantly more expensive with an average cost of $24.59 [SD $14.71] per ounce vs. $6.85 [SD 8.66] for traditional, and $9.38 [SD 8.92] for sheer suncreen.

**Conclusions:**

Sunscreen that is tinted or sheer and compatible with melanated skin is less available and more costly than traditional white sunscreen in beauty and pharmacy chain stores in NYC. Availability and affordability of sunscreen matching the spectrum of skin tones are essential for optimal sun protection and skin health.

## 1 Introduction

Skin cancer is the most common cancer in the United States (US) [1], and its incidence is rising across the world [2]. Of the three types of skin cancer, melanoma is the deadliest due to its ability to spread throughout the body rapidly if not detected and treated early [3]. Predictions for the US estimate there will be 100,640 newly diagnosed cases and 8290 related deaths due to melanoma in 2024 [4]. According to the American Cancer Society [4], White people are 20 times more likely to develop melanoma than Black people. Specifically, they state, “Overall, the lifetime risk of getting melanoma for White people is about 2.6% (or 1 in 38), 0.1% (1 in 1,000) for Black people, and 0.6% (1 in 167) for Hispanic people” [4].

While the incidence of skin cancers in the US is more common among Whites, non-White patients are more likely to experience more severe disease and have a greater risk for mortality due to differing clinical presentations of disease, genetic risk factors, and higher levels of epidermal melanin, a protective pigment in the skin [5]. Among Hispanics, the largest racial/ethnic group in the US comprising 19 % of the total population [6], the rate of melanoma incidence has risen 20 % over the past two decades with Hispanics being younger at the age of diagnosis, more likely to have regional involvement and distant metastasis, and having a worse survival rate compared to non-Hispanic Whites [7]. Black patients are also more likely to present with later-stage melanoma, which in turn is associated with a higher melanoma mortality rate [8].

Skin cancer often results from exposure to ultraviolet (UV) light from the sun or artificial means such as tanning beds. Behaviors such as wearing sunscreen with SPF >30, avoiding tanning beds, seeking shade, and wearing protective clothing and eyewear are prominent recommendations for skin cancer prevention [9]. Sunscreen protects the skin from ultraviolet radiation by blocking UVA and UVB rays. Guidelines for skin cancer prevention state that all individuals, regardless of skin pigmentation, use sunscreen when exposed to harmful UV rays. Despite these well-known recommendations, the use of sunscreen is low overall but, is lowest among persons of color due in large part to the lack of perceived skin cancer threat from UV exposure [10,11].

The Fitzpatrick Skin Phototypes (FSP) scale was created nearly 50 years ago as a measure of the way skin with varying levels of melanin pigment reacts to UV radiation exposure without wearing any sunscreen [12,13]. The FSP scale which spans from I (pale white skin, blue/green eyes, blond/red hair that always burns when exposed to UV light) to VI (dark brown or black skin that never burns when exposed to UV light) has been used in population-based and case-control studies related to UV radiation exposure, tanning and protective behaviors, and skin cancer risk [14]. Lower numbers on the FSP scale have been found to correlate with the development of melanoma and other skin cancers [15].

For those ranking in the lower and mid sections of the FSP scale, the protection from epidermal melanin is decreased, and additional skin protection is warranted. The American Academy of Dermatology states [16], “Yes, people of color should wear sunscreen. Dermatologists recommend that people of color use sunscreen that has broad-spectrum protection, SPF 30 or greater, [and] water resistance.” However, some health professionals find no evidence that individuals ranking highest on the FSP scale should be advised to wear sunscreen [17]. As the debate goes on, it is not clear to people of color what their actual skin cancer risk is and if they should take steps to protect themselves from UV radiation. The confusion around sunscreen use is most apparent in a survey conducted by Consumer Reports [18] that found 61 % of Black respondents and 23 % of Latino respondents never wear sunscreen. Some speculate that misperceptions related to risk drive the underuse of sunscreen among individuals with melanated skin [19,20]. Beyond knowledge of risk and ways to reduce that risk is the availability of products to meet the needs of individuals with melanated skin. Mintel, a market research firm that monitors the sun protection market, reported that 84 % of Black sunscreen users said there are not enough choices on the market for their skin tones [21]. In another study that reviewed websites with recommendations on sunscreen choices for patients of color, compared to those with white or fair skin, chemical sunscreens (70 % vs. 36 %) were often recommended, as were more expensive products (median cost $14 vs. $11 per ounce), despite the lower SPF value [22].

For the current study, we hypothesized that the underuse of sunscreen among persons with melanated skin is, in part, due to a lack of availability (selection of products and cost of products) of sunscreen. In our review of the literature, we did not identify any studies that conducted a thorough, in-person analysis of the availability and pricing of sunscreen for melanated skin. Therefore, the purpose of this study was to determine the availability and pricing of sunscreen for melanated skin at a representative sample of beauty and pharmacy/wellness retail chain stores in New York City.

## 2 Materials and methods

We identified the three largest beauty supply chain stores and the three largest pharmacy, health, and wellness retail chain stores in the US and selected one location of each in or near the Harlem neighborhood of Upper Manhattan. Retail chain stores are comprised of two or more stores that share the same ownership and sell the same lines of goods throughout the enterprise. Beauty supply chain stores sell primarily cosmetics, haircare products, beauty accessories, and personal grooming products whereas pharmacy, health, and wellness chain stores often sell the same goods as beauty supply stores plus prescription and over-the-counter medications overseen by licensed pharmacists and may offer health services such as vaccinations. Harlem is 1.4 mile^2^ in area with 138,953 residents, 46 % of whom are Black and 28 % are Hispanic [23]. Each store was visited in person during May and June of 2023, months during which sunscreen products are sold.

At each store, the number of sunscreen brands for sale was identified. Only products specifically labeled as sunscreen that were in stock (on the shelf) were included in this analysis. For example, makeup that included SPF, tanning oil with no SPF, and products that had inventory tags on the shelf but were not stocked were excluded from this study. The number of products available on the shelf was recorded using a data collection instrument developed specifically for this purpose. Other information gathered included: the product name brand, SPF level, application type (lotion/cream, spray, gel, oil-based, stick, mousse, or other), coloration (with tint, sheer, or traditional – no tint, not sheer), total cost, and weight in ounces. For tinted sunscreen, the number of skin tones was recorded. When tinted sunscreen was identified, the tone displayed on the product was visually matched to the FSP scale [24] to recreate the consumer experience in choosing a tone to match their skin.

### 2.1 Statistics

We calculated the cost per ounce for each product and performed a descriptive analysis of the characteristics of the products that included frequency distributions for categorical variables and mean, standard deviation, and range for continuous variables. Univariable analyses were performed using the chi-square test of association and Analysis of Variance (ANOVA). *P*-values less than 0.5 were considered statistically significant. As this study did not involve human subjects, it was considered exempt from Institutional Board Review. All analyses were conducted using SPSS, version 28 [25].

## 3 Results

A total of 385 sunscreen products were identified throughout the 6 stores visited. Most of the products (*n* = 274, 71.2 %) were identified in the pharmacy, health, and wellness chain stores, whereas 111 (28.8 %) were identified in the beauty supply chain stores (Table 1).

**Table 1:** Comparison of sunscreen characteristics by retail source (beauty supply store vs. pharmacy, health, and wellness store).

	Total	Beauty supply				Pharmacy, health, wellness				*P* value
		Total	Store 1	Store 2	Store 3	Total	Store 1	Store 2	Store 3	
No. products per store	385	111 (28.8)	38 (34.2)	24 (21.6)	49 (44.1)	274 (71.2)	47 (17.2)	79 (28.8)	148 (54.0)	
No. brands per store	51	40	8	12	20	40	7	13	20	
Cost per ounce										<0.001
Mean [SD]	$8.30 [10.06]	$16.65 [13.50]	$10.80 [9.19]	$10.17 [9.57]	$24.36 [14.15]	$4.92 [5.87]	$4.60 [5.78]	$4.54 [4.79]	$5.22 [5.57]	
Range	$0.47–$64.00	$1.94- $64.00	$1.94–$48.00	$2.87–$50.00	$3.78–$64.00	$0.47–$41.49	$0.47–$35.53	$1.25–$35.83	$1.16–$41.50	
SPF										0.003
<30	12 (3.1)	1 (0.9)	1 (2.6)	0 (0.0)	0 (0.0)	11 (4.0)	3 (6.4)	6 (7.6)	2 (1.4)	
30–50	316 (82.1)	102 (91.9)	32 (84.2)	22 (91.7)	48 (98.0)	214 (81.8)	32 (68.1)	62 (78.5)	120 (81.1)	
>50	57 (14.8)	8 (7.2)	5 (13.2)	2 (8.3)	1 (2.0)	6 (2.2)	12 (25.5)	11 (13.9)	26 (17.6)	
Median	50	40	35	30	45	50	50	50	50	
Range	15–110	20–70	20–70	30–60	30–70	15–110	15–110	15–70	15–100	
Application Type										<0.001
Lotion/cream	226 (58.7)	82 (73.9)	24 (63.2)	16 (66.7)	42 (85.7)	144 (52.6)	26 (55.3)	41 (51.9)	77 (52.0)	
Spray	122 (31.7)	18 (16.2)	10 (26.3)	5 (20.8)	3 (6.1)	104 (38.0)	19 (40.4)	32 (40.5)	53 (35.8)	
Gel	6 (1.6)	4 (3.6)	1 (2.6)	0 (0.0)	3 (6.1)	2 (0.7)	0 (0.0)	0 (0.0)	2 (1.4)	
Oil-based	5 (1.3)	4 (3.6)	2 (5.3)	2 (8.3)	0 (0)	1 (0.4)	0 (0.0)	0 (0.0)	1 (0.7)	
Stick	18 (4.7)	2 (1.8)	1 (2.6)	0 (0.0)	1 (2.0)	16 (5.8)	2 (4.3)	2 (2.5)	12 (18.2)	
Mousse	2 (0.5)	1 (0.9)	0 (0.0)	1 (4.2)	0 (0.0)	1 (0.4)	0 (0.0)	0 (0.0)	1 (0.7)	
Other	6 (1.6)	0 (0.0)	0 (0.0)	0 (0.0)	0 (0.0)	6 (2.2)	0 (0.0)	4 (5.1)	2 (1.4)	
Coloration										<0.001
Traditional white	303 (78.7)	79 (71.2)	31 (81.6)	22 (91.7)	26 (53.1)	224 (81.8)	34 (72.3)	72 (91.1)	118 (79.7)	
Sheer	59 (15.3)	15 (13.5)	5 (13.2)	2 (8.3)	8 (16.3)	44 (16.1)	12 (25.5)	5 (6.3)	27 (18.2)	
Tinted	23 (6.0)	17 (15.3)	2 (5.3)	0 (0.0)	15 (30.6)	6 (2.2)	1 (2.1)	2 (2.5)	3 (2.0)	
Number of tones offered										0.43
1	19 (82.6)	15 (88.2)	2 (100.0)	0 (0.0)	13 (86.7)	4 (66.7)	0 (0.0)	1 (50.0)	3 (100.0)	
2	1 (4.3)	0 (0.0)	0 (0.0)	0 (0.0)	0 (0.0)	1 (16.7)	0 (0.0)	1 (50.0)	0 (0.0)	
3	2 (8.7)	1 (5.9)	0 (0.0)	0 (0.0)	1 (6.7)	1 (16.7)	1 (100.0)	0 (0.0)	0 (0.0)	
4	1 (4.3)	1 (5.9)	0 (0.0)	0 (0.0)	1 (6.7)	0 90.0)	0 (0.0)	0 (0.0)	0 (0.0)	

**Fig. 1:**
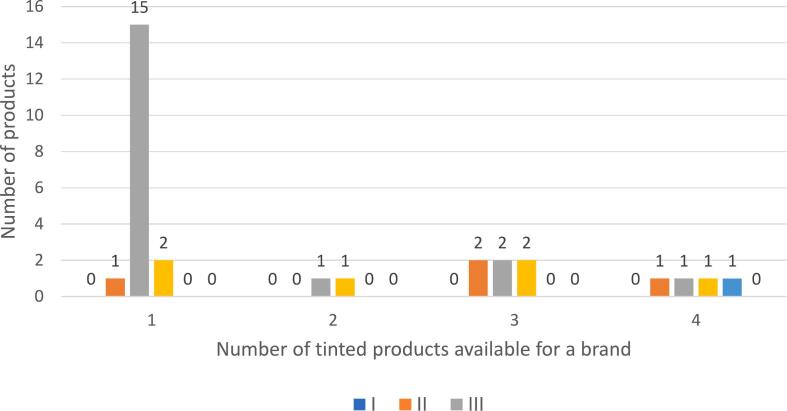
Comparison of sunscreen brands by number of tinted products and Fitzpatrick Skin Phototype matching.

In all, 51 separate brands were identified, ranging from 8 to 20 brands in the beauty supply stores and 7–20 brands in the pharmacy health and wellness stores. The average cost per ounce of all sunscreen products was $8.30 [SD $10.06] (range $0.47–$64.00 per ounce), with beauty supply store offerings costing significantly more than the pharmacy, health, and wellness store offerings ($16.65 vs. $4.92, *p* < 0.001). Overall, SPF coverage of products ranged from 15 to 110 but the overwhelming majority (*n* = 316, 82 %) of products were in the SPF range of 30–50. Median SPFs were lower in beauty supply stores (30–45 vs. 50, *p* = 0.003). There was a significant difference (*p* < 0.001) in the distribution of sunscreen application types between pharmacy, health, and wellness and beauty store offerings. The most commonly found application method in all products was lotion (*n* = 226, 58.7 %), followed by spray (*n* = 122, 31.7 %) and stick (*n* = 18, 4.7 %) with spray application more frequently sold in the pharmacy, health, and wellness stores (38.0 % vs. 16.2 %). Traditional white sunscreen products were abundantly available for purchase overall (*n* = 303, 78.7 %) followed by sheer (*n* = 59, 15.3 %) and tinted (*n* = 23, 6.0 %) products. Beauty supply stores offered more tinted sunscreen options than did pharmacy, health, and wellness stores (*n* = 17,15.3 % vs. *n* = 6, 2.2 %, *p* < 0.001). Of the tinted sunscreen products, 19 brands offered a single tone (FSP II through IV) and 4 brands had multiple shades to match skin tones (FSP II through V) (Fig. 1). None of the products matched FSP VI, the darkest skin tone. There was no statistically significant difference in the number of tinted sunscreen products between the two types of stores (*p* = 0.43).

Table 2 demonstrates a comparison of sunscreen products by coloration (traditional, sheer, and tinted). There were statistically significant differences between store type and their product coloration offerings (*p* < 0.0001). Compared to pharmacy, health, and wellness stores, beauty supply stores more often stocked tinted sunscreen products (73.9 % vs. 26.1 %) whereas pharmacy, health, and wellness stores more often made traditional and sheer sunscreen available (73.9 % and 74.6 % vs. 26.1 % and 25.4 %, respectively). Of all sunscreen coloration types studied, tinted sunscreen was significantly more expensive with an average cost of $24.59 [SD $14.71] per ounce (range $1.86- $64.00 per ounce). All tinted sunscreen products had SPF ranging between 30 and 50 which differed significantly from the traditional and sheer products with SPF <30 to >50 (*p* = 0.02).

**Table 2:** Comparison of sunscreen products by coloration (traditional, sheer, and tinted).

	Total	Traditional (n = 303, 78.7 %)	Sheer (n = 59, 15.3 %)	Tinted (*n* = 23, 6.0 %)	P value
Type of retail chain					<0.001
Beauty supply	111 (28.8)	79 (26.1)	15 (25.4)	17 (73.9)	
Pharmacy, health, wellness	274 (71.2)	224 (73.9)	44 (74.6)	6 (26.1)	
Cost per ounce					<0.001
Mean	$8.30 [10.06]	$6.85 [8.66]	$9.38 [8.92]	$24.59 [14.71]	
Range	$0.47–$64.00	$0.47–$55.00	$1.75–$42.86	$1.86–$64.00	
SPF					0.02
<30	12 (3.1)	10 (3.3)	2 (3.4)	0 (0.0)	
30–50	316 (82.1)	249 (82.2)	44 (74.6)	23 (100.0)	
>50	57 (14.8)	44 (14.5)	13 (22.0)	0 (0.0)	

## 4 Discussion

In our study to observe the availability and cost of sunscreen for individuals with melanated skin in beauty supply and pharmacy, health, and wellness chain stores in Upper Manhattan, we found both a lack of availability and affordability of sunscreen products for melanated skin. Of the 51 unique brands of sunscreen representing 385 separate products, 82 (21.5 %) were sheer or tinted and thus appropriate for melanated skin. The majority of products targeting individuals with varying tones of melanated skin (73.9 %) were sold in beauty supply stores with a mean cost per ounce of $24.59 [SD $14.71] for tinted sunscreen and $9.38 [SD $8.92] for sheer compared to $6.85 [SD $8.66] for traditional sunscreen.

Non-white individuals are at higher risk for more severe disease and greater risk for mortality due to melanoma but not all healthcare providers agree that protection from harmful solar UV radiation can reduce this risk [[5], [6], [7], [8],^17^]. It is important to note that regardless of one's skin tone, all people are susceptible to damage from UV radiation, hyperpigmentation, increased skin damage (e.g., wrinkles and age spots) [26] and melanin is not impenetrable by all UV rays [27]. Recommendations of the American Academy of Dermatology [16] include that people of color should wear sunscreen. They define people of color to include “diverse skin colors and includes people of African, Asian, Latino, Mediterranean, Middle Eastern, and Native American descent.” Consistent with this, there is a call for products that are inclusive of this level of diversity. In the short term, without agreement among medical personnel about the necessity of sun protection for melanated skin, provider recommendations to protect melanated skin from the sun will continue to be inconsistent, and consumer awareness of risk and demand for these products will likely remain low. In the long term, the lack of recommendations for and use of sunscreen and protection from the sun among individuals of color has the potential to contribute to the differential burden of disease and exacerbate health disparities.

Physician recommendation for nearly all health behaviors is perhaps the most important predictor of patient compliance [28]. It is therefore reasonable to suggest that inconsistent health risk management messaging from providers has resulted in the perpetuation of perceptions among the public that skin of color is immune to the harmful effects of solar UV radiation, including the risk of skin cancer. Low-risk awareness and lack of perceived need likely drive consumer demand for sunscreen products to protect melanated skin. This may in turn underly the very limited product availability and high cost of sunscreen for melanated skin. The limited availability of products for this group in major retail stores potentially reinforces individual perceptions that these products are unnecessary. The higher than traditional sunscreen cost of these products may further imply to some that sunscreen for melanated skin is a luxury item intended for only those with relative wealth and status [29].

We identified only a single study that examined the availability and cost of sunscreen based on skin color. Song et al. [22] conducted a Google search of sunscreen products using search terms indicating a spectrum of skin tones from dark and black to fair and white. The search was limited to the first page of results in Google. An anonymous survey of dermatologic practitioners associated with the tertiary institutions affiliated with Harvard Medical School in Boston was then administered. Of the 14 unique websites evaluated, 88 distinct sunscreen products were identified with 20 (22.7 %) recommended for people of color. Most commonly, the sunscreen products for people of color were chemical or chemical/physical in nature with a median SPF of 32.5 and a median cost of $14 per ounce. Results of the survey of dermatologists showed that they counseled patients with darker skin tones 31.1 %, 46.8 %, 18.2 %, and 3.9 % “never,” “sometimes,” “most of the time,” and “always,” respectively. By comparison, our study evaluated 385 products sold at major chain retailers, 21.3 % of which were suited for persons with darker skin tones – demonstrating a similar proportion of products for individuals with melanated skin. In our study, we found a mean cost per ounce of sheer sunscreen products of $9.38 (*n* = 59) and $24.59 (*n* = 23) for tinted sunscreen with a computed cost per ounce for the combined sheer and tinted products of $13.65, again comparable to that of Song et al. as they did not differentiate between the two sunscreen types. These findings support that our observations may represent a pattern of limited availability and accessibility/affordability of sunscreen compatible with melanated skin, at least in northeast United States.

Rectifying the issues surrounding sunscreen use among melanated people is complex and multilevel. The role of UV radiation exposure and its relationship to the development of melanoma among individuals with darker skin tones relies on the presentation of scientific evidence followed by clear medical guidelines. Both providers and patients need to understand the risk and how to reduce it, a task that demands the dissemination of information through appropriate channels by reputable sources. Lastly, manufacturers and distributors of sun protection products for people of color need to develop and market these products such that they are available and affordable.

Current market research shows some reason for optimism. A study of 1,200 customer reviews on the Sephora website evaluating consumer preferences when choosing a sunscreen product found that 62 % (32/51) of products provided only one shade which is consistent with our findings, and that tone incompatibility, especially among consumers with dark skin tones, was the most commonly cited criticism of tinted sunscreen products [30]. Furthermore, in a recent research report on the health and wellness market in the U.S, McKinsey and Company, a global management consulting firm, reported that expenditures on wellness products and services in the US now exceed $450 billion and are growing at more than 5 % annually [31]. However, the needs of millennial consumers and Black consumers are not sufficiently being met. The report cites that 47 %–55 % of Black consumers wanted more wellness products to meet their needs compared to 30 %–35 % of White consumers.

We must also acknowledge certain limitations of our study. While observational in design using a small convenience sample, we intentionally examined in-person product availability to mirror a consumer experience in real-time and conducted our study in stores that were affiliated with large conglomerates where product availability tends to be mainstream. Our findings may not be generalized geographically both by price and availability. Despite these shortcomings, this study fills an important gap in the literature.

## 5 Conclusions

Traditionally, sunscreens contain sun-reflective ingredients that create a white cast which is not desirable for persons with melanated skin. Our research indicates that there is still work to be done to increase the availability and affordability of sunscreens that are sheer or have a wide variety of tones. Affordable sunscreen that matches a variety of skin tones is essential for skin health and can influence skin cancer prevention efforts thereby reducing disparities in prevention efforts. Future research in this arena should focus on the extent to which melanin can protect against UV radiation [32], the most effective ways to measure skin tone in a diverse population [33,34], inconsistency in provider recommendations to protect melanated skin [22], potential implicit bias in dermatology training related to skin cancer that results in fewer sunscreen and skin protection recommendations for patients of color [35], and consumer confusion related to conflicting messaging related to sunscreen and its efficacy for melanated skin.

## Funding sources

This research did not receive any specific grant from funding agencies in the public, commercial, or not-for-profit sectors.

## CRediT authorship contribution statement

**Emerson D. Basch:** Conceptualization, Data curation, Methodology, Project administration, Writing – original draft. **Grace C. Hillyer:** Data curation, Formal analysis, Methodology, Visualization, Writing – review & editing.

## Declaration of competing interest

The authors declare that they have no known competing financial interests or personal relationships that could have appeared to influence the work reported in this paper.
